# Recent Advances in Crystalline Oxidopolyborate Complexes of *d*-Block or *p*-Block Metals: Structural Aspects, Syntheses, and Physical Properties

**DOI:** 10.3390/molecules26133815

**Published:** 2021-06-22

**Authors:** Shu-Sheng Xin, Ming-Hua Zhou, Michael A. Beckett, Chun-Yang Pan

**Affiliations:** 1 School of Chemical Engineering and Light Industry, Guangdong University of Technology, Guangzhou 510006, China; xinshusheng0604@163.com (S.-S.X.); zmh1277502231@163.com (M.-H.Z.); 2 School of Natural Sciences, Bangor University, Bangor LL57 2UW, UK

**Keywords:** borates, coordination compounds, inorganic–organic hybrids, oxidoborate ligands

## Abstract

Crystalline materials containing hybrid inorganic–organic metal borates (complexes with oxidoborate ligands) display a variety of novel framework building blocks. The structural aspects of these hybrid metallaoxidoborates containing Cd^(II)^, Co^(II)^, Cu^(II)^, Ga^(III)^, In^(III)^, Mn^(II)^, Ni^(II)^ or Zn^(II)^ metal centers are discussed in this review. The review describes synthetic approaches to these hybrid materials, their physical properties, their spectroscopic properties and their potential applications.

## 1. Introduction

In borate chemistry (n.b. oxidoborate is the recommended IUPAC name for oxidized oxygen containing borates [1]) the boron centers are bound to oxygen atoms as *sp*^2^ hybridized triangular {BO_3_} (Δ) or *sp*^3^ hybridized {BO_4_} tetrahedral (*T*) structural units [2,3,4]. These fundamental units can be aggregated, employing organic cations or transition-metal cations as templating agents [5], with condensation and oxygen atom corner sharing into larger oxidoborate clusters. In these structures, terminal oxygen atoms (i.e., those not corner shared) are generally also bound to hydrogen atoms as hydroxy groups [2,3,4]. Such oxidoborates are often described as hydrated borates [6] and these compounds are readily formed under relatively mild conditions [2,3,4,5,6]. Harsher conditions can lead to anhydrous borates and although very rare, even to the possibility of edge sharing oxygen atoms [7]. Descriptors have been developed by Christ and Clark [2] and by Burns and co-workers [4] to designate reoccurring structural motifs as framework building blocks (FBBs) and Christ and Clark’s descriptors [2] are used in this review. Hydrated oxidiborates may enter the primary coordination shell of metals, with formation of *O*-donor coordinate bonds with the result of even more complex and diverse species. Coordination compounds containing oxidopolyborate ligands are therefore an important sub-class of synthetic oxidoborate compounds and recent progress in this area is the subject of this review. Since insular oxidopolyborate anions partnered by cationic transition-metal complexes do not contain oxidoborates as *ligands* they are not within the scope of this review and are excluded. Metal complexes often contain more than one ligand type and those that contain oxidoborate ligands and conventional organic ligands with typical donor atoms may also be classified as inorganic–organic hybrid materials [8,9]. Such hybrid compounds may potentially have unique and useful properties as a result of combining and/or enhancing properties associated with non-hybrid single materials.

This review is designed to be comprehensive within the defined topic and covers recently reported (twenty-first century) literature. It reports on structural chemistry, synthetic methods, physical properties and possible applications and focusses on complexes containing oxidoborate ligands. The following *d*-block and *p*-block metal ions form such complexes: Cd^(II)^, Co^(II)^, Cu^(II)^, Ga^(III)^, In^(III)^, Mn^(II)^, Ni^(II)^ and Zn^(II)^ and subsections are dedicated to each metal. Oxidoborate ligands are discussed by increasing boron number within these metal borate subsections. Compounds containing oxidoborates ligands were first reported in the twentieth century and for information on such compounds the reader is referred to an earlier review [3] which surveys general structural aspects of oxidoborate chemistry.

## 2. Structural (XRD) Studies

### 2.1. Cadmium(II) Borates

Several oxidoborate coordination compounds of Cd^(II)^ have been reported during the review period: [Cd(dab)_0.5_(dab’)_0.5_{B_5_O_7_(OH)_3_}]_n_ (**1**) (dab = 1,4-diaminobutane) [8], [Cd(dap)_0.5_(dap’)_0.5_{B_5_O_8_(OH)}]_n_·nH_2_O (**2**) (1,3-diaminopropane) [9], [Cd(tren){B_8_O_11_(OH)_4_}]_n_ (**3**) (tren) = tris(2-aminoethyl)amine) [10], [Cd(pn){B_6_O_7_(OH)_6_}]_n_·nH_2_O (**4**) (pn = 1,2-diaminopropane) [11], [{Cd_3_(H_2_O)_4_(NO_3_)_2_}{B_6_O_9_(OH)_2_}_2_]_n_ (**5**) [12] and four related compounds, exemplified by [pyH]_2_[Cd(py)_2_{B_14_O_20_(OH)_6_}](**6**) (py = pyridine) [13].The FBB’s in these compounds are mostly complex with the pentaborate(2-) unit in **1** is based on a FBB of 4(4-1) with a pendant -B(OH)_2_ unit (FBB = 1) replacing a terminal H atom on a *T* unit of the larger FBB and can be designated a compounded descriptor 5:[4:(2Δ+2*T*)+Δ]. The FBB for **2** is based on the pentaborate(2-) unit 5:(3Δ+2*T*). The octaborate(2-) and the tetradecaborate(4-) FBBs in **3** and **6** are also best designated as compounded descriptors 8:[5:(4Δ+*T*)+3:(2Δ+*T*)] and 14:[{7:(5Δ+2*T*)}_2_] respectively, with the latter dimer based on a FBB unit of 7. The condensed hexaborate(2-) units in **4** and **5** have a more standard descriptor based on a FBB unit of 6 i.e., 6:(2Δ+3*T*).

**Figure 1:**
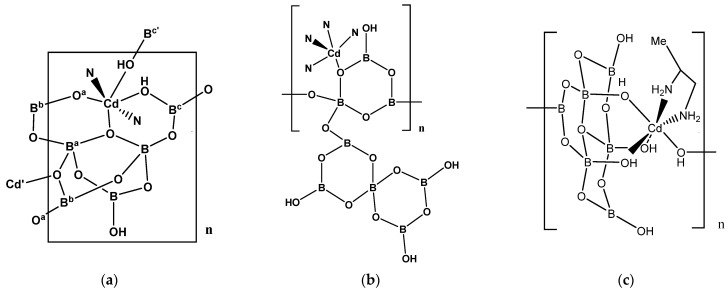
The building units of (**a**) [Cd(dab)_0.5_(dab’)_0.5_{B_5_O_7_(OH)_3_}]_n_ (**1**), (**b**) [Cd(tren){B_8_O_11_(OH)_4_}]_n_ (**3**), and (**c**) [Cd(pn){B_6_O_7_(OH)_6_}]_n_·nH_2_O (**4**).

Compound **1** is a hybrid inorganic–organic 3-D coordination polymer [8]. The 22-electron Cd^(II)^ center adopts a distorted six-coordinate octahedral complex with two *trans N*-donor from two different 1,4-dab ligands (forming a 1-D chain) and four *O*-donor atoms from two oxidoborate network ligands (Figure 1a).

Compound **2** is also a hybrid inorganic–organic 3-D coordination polymer [9]. In **2** the 2-D layers of repeating neutral [Cd{B_5_O_8_(OH)}] units are linked by bridging dap units into a 3-D structure, with each 20-electron Cd^(II)^ center being 5-coordinate and ligated by two monodentate *N*-donor dap ligands and three *O*-donors from the oxidoborate framework.

Compound **3** is a Cd^(II)^ complex comprised of an anionic oxidoborate ligand {B_8_O_11_(OH)_4_}^2−^ fragment coordinated to a supporting {Cd(tren)}^2+^ fragment (Figure 1b) [10]. The 20-electron Cd^(II)^ center in **3** is five-coordinate and is coordinated by four *N*-donors from the tren ligand and one *O*-donor from the bridging O atom from the {B_3_O_6_(OH)} sub-unit of the octaborate(2-) anion. These {B_3_O_4_(OH)} sub-units, with additional pendant {B_5_O_7_(OH)_3_} sub-units, link together into a 1-D chain polymer. These 1-D chains are joined together into a 3-D framework via extensive H-bond interactions.

Compound **4** is an octahedral 22-electron Cd^(II)^ complex with a *cis*-pn ligand and four *O*-donors from two hexaborate(2-) ligands, forming a 1-D coordination polymer chain [11]. One of the hexaborate ligands is coordinated *fac* to the Cd^(II)^ center via the three OH groups bound to tetrahedral boron atoms and the final coordination bond is formed from a trigonal boron OH group from another hexaborate(2-) unit (Figure 1c). The structure of this complex is described as [Cd(1,2-dap)][B_6_O_11_(OH)_2_]·H_2_O in [11] but is better formulated as [Cd(pn){B_6_O_7_(OH)_6_}]_n_·nH_2_O (**4**). The hexaborate(2-) units in **4** not only bridge two Cd^(II)^ centers but are also further linked together to form an extended network which is interconnected via a strong H-bonding.

**Figure 2:**
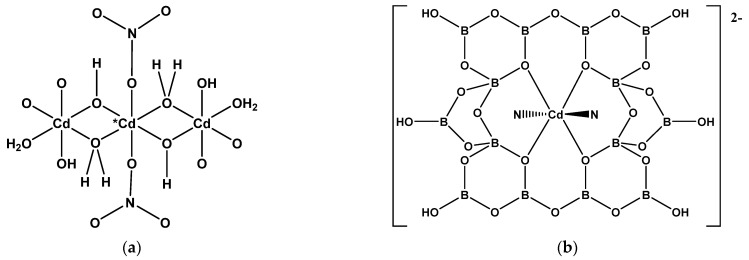
(**a**) The cationic {Cd_3_(H_2_O)_4_(NO_3_)_2_}^4+^ unit of [(Cd_3_){B_6_O_9_(OH)_2_}_2_(NO_3_)_2_(H_2_O)_4_]_n_ (**5**). Donor atoms marked O and OH from oxidohexaborate clusters, and (**b**) the building unit of Cd^(II)^-centered metallaoxidoborate cluster anion, *trans*-[Cd(py)_2_{B_14_O_20_(OH)_6_}]^2−^ (**6**).

Compound **5** has a unique structure, and it is comprised of 2-D planes of condensed {B_6_O_9_(OH)_2_}^2−^ units coordinated to the terminal 22-electron Cd^(II)^ centers of a linear {Cd_3_(H_2_O)_4_(NO_3_)_2_}^4+^ unit (Figure 2a) is such a way as to form a crosslinked 3-D network [12]. The central 22-electron Cd^(II)^ center of the centrosymmetric {Cd_3_(H_2_O)_4_(NO_3_)_2_}^4+^ sub-unit (marked Cd* in Figure 2a) has two *trans* NO_3_^−^ ligands and all three Cd^(II)^ centers are octahedral with remaining sites occupied by terminal or bridging H_2_O ligands or oxidoborate *O*-donor centers.

Compound **6** is typical of a series of four hybrid Cd^(II)^ oxidoborates that exhibit 3-D open-framework with novel topologies [13]. All the networks are comprised of a novel Cd^(II)^ centered complex *trans*-[Cd(py)_2_{B_14_O_20_(OH)_6_}]^2−^ and there are further interionic links via H-bonding interactions. As shown in Figure 2b, the [B_14_O_20_(OH)_6_]^4−^ coordinates by four *O*-donors from the four boroxole {B_3_O_3_} rings to the Cd^(II)^ center in a square planar arrangement, with two additional axial *N*-donor ligands, resulting in a 22-electron octahedral complex.

### 2.2. Cobalt(II) Borates

**Figure 3:**
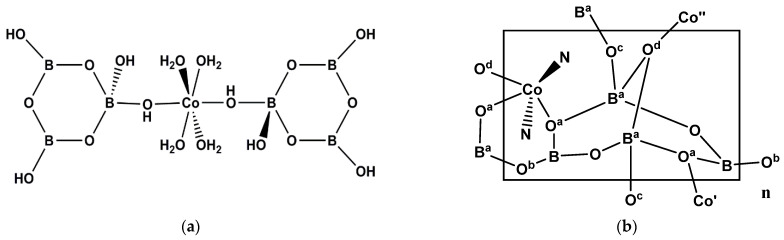
(**a**) The structure of [Co(H_2_O)_4_{B_3_O_3_(OH)_4_}_2_] (**7**), and (**b**) the building unit of [Co(dap)_0.5_(dap’)_0.5_{B_4_O_7_}]_n_ (**8**).

The triborate(1-) ligand with the FBB unit of a 6-membered boroxole ring with two {BO_3_} units and one {BO_4_} unit i.e., 3:(2Δ+*T*) (Figure 3a) is present in the recently synthesized salt [Co(H_2_O)_6_]_2_[NO_3_]_2_·[Co(H_2_O)_4_{B_3_O_3_(OH)_4_}_2_]·2H_2_O (**7**) [14]. The 19-electron Co^(II)^ centers are both octahedral and the two [B_3_O_3_(OH)_4_]^−^ ligands in the neutral [Co(H_2_O)_4_{B_3_O_3_(OH)_4_}_2_] complex are *trans* and are coordinated by hydroxy *O*-donors bound to tetrahedral boron atoms.

A diagram of the structure of [Co(dap)_0.5_(dap’)_0.5_{B_4_O_7_}]_n_ (**8**) [8] (Figure 3b) shows a five-coordinate trigonal-bipyramidal coordination geometry at the 17-electron Co^(II)^ center with two *N*-donor coordinate bonds, from two different dap ligands and forming a 1-D chain, and three *O*-donor atoms from three anionic {B_4_O_7_}^2−^ tetraborate units. The Christ and Clark descriptor for these units with a FBB of 4 is 4-1:(2Δ+2*T*).

**Figure 4:**
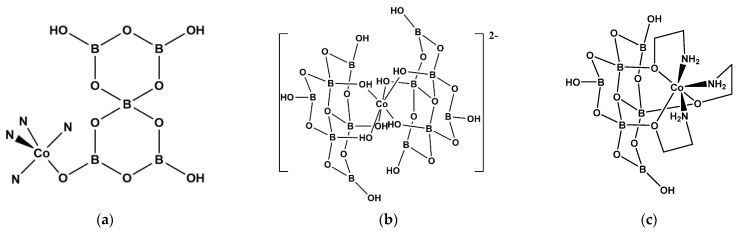
Drawings of (**a**) the uncharged unit of [Co(tren) {OB_5_O_6_(OH)_3_}] (**9**), (**b**) the dianion observed in [1-cyanopiperazinium][Co{B_6_O_7_(OH)_6_}_2_]·4H_2_O (**11**), and (**c**) the structure of [Co{(NH_2_CH_2_CH_2_O)_3_B_6_O_7_(OH)_3_}] (**12**).

The basic building unit of [Co(tren){OB_5_O_6_(OH)_3_}] (**9**) is shown in Figure 4a [15]. The [OB_5_O_6_(OH)_3_]^2−^ ligand can be described as 5:(4Δ+*T*) with one of four trigonal units deprotonated. The 17-electron Co^(II)^ center exhibits a trigonal-bipyramidal coordination geometry, with four *N*-donor atoms and one *O*-donor atom. Compound **9** forms 3-D supramolecular network through extensive H-bond interactions.

The homoleptic bis(hexaborate(2-))cobalt(II) complexes [piperazine-1,4-dium] [Co{B_6_O_7_(OH)_6_}_2_]·6H_2_O (**10**) [16] and [1-cyanopiperazinium][Co{B_6_O_7_(OH)_6_}_2_]·4H_2_O (**11**) [17] have been recently synthesized and characterized crystallographically. The oxidoborate ligands in **10** and **11** are designated 6:(3Δ+3*T*) and it is the three hydroxyl *O* atoms on the three {BO_4_} centers that coordinate in *fac*- geometries to the octahedral 19-electron Co^(II)^ centers (Figure 4b). In both these structures there are strong templating interionic H-bond interactions.

The derivatized hexaborate(2-) ligand observed in [Co{(NH_2_CH_2_CH_2_O)_3_B_6_O_7_(OH)_3_}] (**12**) [18] has three 2-amino ethoxy groups in place of the three -OH groups on the tetrahedral boron centers of a hexaborate(2-) ion found in **10** and **11**. It functions as a hexadentate ligand through the three *O*-donors, bound to the aminoethyl substituents and the three amino *N*-donors to the 19-electron Co^(II)^ center (Figure 4c).

### 2.3. Copper(II) Borates

Oxidoborates coordinated to Cu^(II)^ centers are not uncommon and are available for diborate(2-), pentaborate(1-), hexaborate(2-) and icosaborate(12-) anions. The diborate(2-) anion is observed as part of the templated oxidoborate found in [H_3_O]_4_[Cu_7_(NH_3_)_2_(H_2_O)_4_{B_24_O_39_(OH)_12_}]·13H_2_O (**13**) [19] and this will be discussed later in this sub-section.

**Figure 5:**
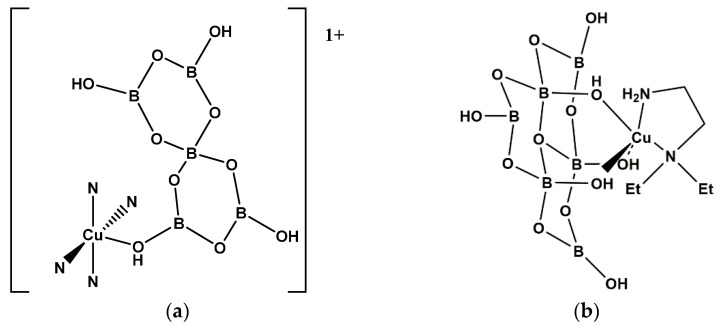
(**a**) The cation present in [Cu(pn)_2_{B_5_O_6_(OH)_4_}][B_5_O_6_(OH)_4_]·4H_2_O (**14**), and (**b**) the uncharged building unit of [Cu(deen){B_6_O_7_(OH)_6_}]·5H_2_O (**19**).

As shown in Figure 5a, [Cu(pn)_2_{B_5_O_6_(OH)_4_}][B_5_O_6_(OH)_4_]·4H_2_O (**14**) [20] is an ionic compound comprised of a cationic hybrid Cu^(II)^ complex containing a pentaborate(1-) ligand based on a FBB of 5:(4Δ+*T*). This +1 cation is partnered with an additional insular [B_5_O_6_(OH)_4_]^−^ anion. The 19-electron Cu^(II)^ ion in **14** has a distorted square-based pyramidal geometry with four *N*-donor atoms, and an axial *O*-donor pentaborate(1-) with a *T^5^* [21] of 0.87. A sixth O-donor H_2_O potential ‘ligand’ is axially *trans* to the pentaborate(1-) ligand, but the Cu-O distance is not within normal (or even long) bonding distances.

Coordinated hexaborate ligands are well represented in Cu^(II)^ coordination chemistry as illustrated by the following examples: [Cu(NH_3_)_2_{B_6_O_7_(OH)_6_}]_n_·2nH_2_O (**15**) [22], [Cu(en){B_6_O_7_(OH)_6_}]_n_·3nH_2_O (**16**) (en = 1,2-diaminoethane) [23], [Cu(dmen){B_6_O_7_(OH)_6_}]·4H_2_O (**17**) (dmen = *N*,*N*-dimethyl-1,2-diaminoethane) [20], [Cu(tmeda){B_6_O_7_(OH)_6_}]·6H_2_O (tmeda = *N*,*N*,*N’*,*N’*-tetramethyl-1,2-diaminoethane) (**18**) [20], and [Cu(deen){B_6_O_7_(OH)_6_}]·5H_2_O (**19**) (deen = *N*,*N*-diethyl-1,2,-diaminoethane) [22]. The organic *N*-donor ligands in **17**, **18** and **19** are relatively sterically demanding and the resulting neutral coordination complexes have square-based pyramidal 5-coordinate 19-electron Cu^(II)^ geometries. Each Cu^(II)^ center is coordinated by two *N*-donors and three *O*-donors from the oxidohexaborate(2-) ligands. This is illustrated in Figure 5b for **19**. The organic *N*-donor ligands in **15** and **16** are relatively small and this permits the Cu^(II)^ centers to adopt 6-coordinate tetragonally distorted octahedral geometries with the formation of additional Cu-O coordinate bonds from bridging oxidoborate ligands, in a similar way to that observed in the Cd^(II)^ complex, **4**. These 21-electron Cu^(II)^ centers are coordinated by two *N*-donors ligands, three *O*-donors from the *fac*-hexaborate(2-) ligand with a sixth site from an *O*-donor of an ‘adjacent’ hexaborate(2-) by formation of a 1-D coordination polymeric chain.

**Figure 6:**
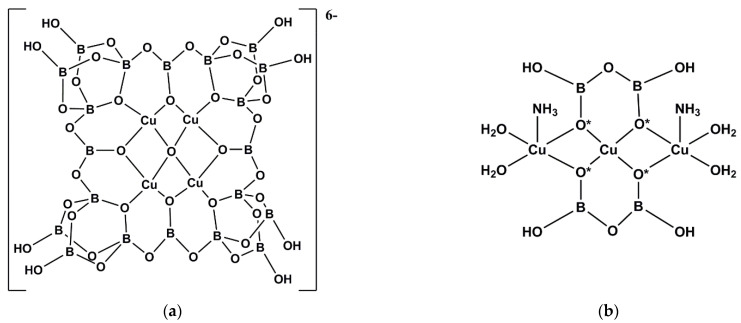
Drawing of (**a**) the structure of the (oxidotetracopper)oxidoicosaborate(6-) anion, [Cu_4_O{B_20_O_32_(OH)_8_}]^6−^, found in **13**, **20** and **21**, and (**b**) the ‘top layer’ of **13** which is comprised of a linear {Cu_3_}^6+^ chain and two coordinated oxidodiborate(2-), [B_2_O_3_(OH)_2_]^2−^, anions. The four O* atoms also bridge to the four Cu atoms in the lower level of **13**.

Three new examples of Cu^(II)^ complexes containing oxidoicosaborate(12-) ligands have been prepared: [H_3_O]_4_[Cu_7_(NH_3_)_2_(H_2_O)_4_{B_24_O_39_(OH)_12_}]·13H_2_O (**13**) [19], H_6_[Cu_4_O{B_20_O_32_(OH)_8_}]·25H_2_O (**20**) [24] and H_6_[Cu_4_O{B_20_O_32_(OH)_8_}]·34H_2_O·8B(OH)_3_ (**21**) [24]. These three compounds are fundamentally structurally identical to Cu^(II)^/oxidoicosaborate(6-) complexes HM_5_[Cu_4_O{B_20_O_32_(OH)_8_}]·32H_2_O (M = Na, K) first synthesized by Heller and described in his early (1986) borate structural chemistry review [3]. The structure of the anion in **13**, **20** and **21** is drawn in Figure 6. It is best described as comprised on four square planar 17-electron Cu^(II)^ ions and a central μ_4_-O^2−^ ion supporting and surrounded by large oxidoicosaborate(12-) ring structure. This ring structure itself is ‘tetrameric’ with four alternating FBB’s of {B1} and {B4} sub-units linked into a larger 24-membered ring with a compound designation of 20:{4:(2Δ+2*T*)+Δ}_4_. Compound **21** has 8 additional B(OH)_3_ molecules per oxidoicosaborate(12-) moiety, and these are situated within channels which are available within the giant structure formed by close-packing the large multi-metallic oxidoborate anions [24].

Compound **13** is unique and contains seven Cu^(II)^ centers. It can be considered to be a large anion based on two ‘layers’: the ‘lower layer’ is as is illustrated in Figure 6a and this layer supports a linear {Cu_3_}^6+^ unit in an ‘upper layer’. The {Cu_3_}^6+^ unit is further supported by two peripheral diborate(2-) anions, and all seven 19-electron Cu^(II)^ centers are 5-coordinate square-based pyramids. This ‘top layer’ of **13** is illustrated in Figure 6b. Structurally, compound **13** has another unusual feature: the presence of B-O^−^ groups. It is very rare for oxidiborates prepared and crystallized from aqueous solution to display B-O^−^ groups arising from trigonal B centers [2]. It is also interesting to note that all such B-O^−^ groups in the ‘upper layer’ of **13** are found bridging two (μ_2_-) or three (μ_3_-) Cu^(II)^ centers and that the central O^2−^ ion bridges five (μ_5_-) Cu^(II)^ centers [19]. There are also four B-O^−^ groups in the ‘lower layer’ of **13**, and in **20** and **21** that each bridge (μ_2_-) two Cu^(II)^ centers.

### 2.4. Gallium(III) and Indium(III) Borates

**Figure 7:**
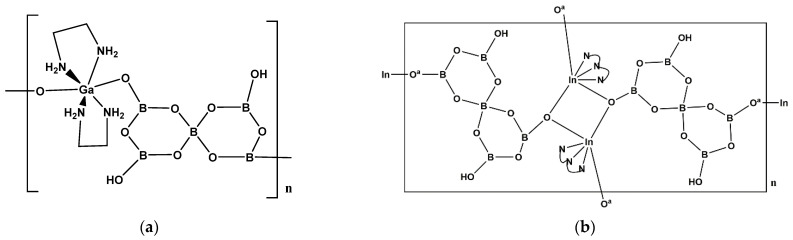
The building unit of (**a**) [Ga(en)_2_{B_5_O_8_(OH)_2_}]_n_·nH_2_O (**22**), and (**b**) the dimeric In^(III)^ centers in [In(dien){B_5_O_8_(OH)_2_}]_n_ (**27**).

Ga^(III)^ and In^(III)^ borates are conveniently considered together. The hybrid oxidoborate, [Ga(en)_2_{B_5_O_8_(OH)_2_}]_n_·nH_2_O (**22**) was first synthesized in 2012 [25]. The two en *N*-donor bidentate ligands occupy four coordination sites around an octahedral 22-electron Ga^(III)^ center and the two remaining *cis*- coordination sites are connected to two different {B_5_O_8_(OH)_2_}^3−^ anions forming a 1-D chain structure (Figure 7a). Two recently reported compounds [Ga(teta){B_5_O_8_(OH)_2_}]_n_·nH_2_O (**23**) (teta = tetraethylenetriamine) and [In(teta){B_5_O_8_(OH)_2_}]n·1.5nH_2_O (**24**) have similar structures [11]. Compound **22** was also reported in 2013 together with three other related compounds: [In(en)_2_{B_5_O_8_(OH)_2_}]·H_2_O (**25**), [In(dap)_2_{B_5_O_8_(OH)_2_}]·H_2_O (**26**), and [In(dien){B_5_O_8_(OH)_2_}]_n_ (**27**) [26]. The structures of **25** and **26** are essentially the same as **22** with a change of metal (to In^(III)^ in **25**) or ligand and metal (to In^III^ and dap in **26)**. Compounds **22**–**27** contain {B_5_O_8_(OH)_2_}^3−^ anions and these anions are based on the frequently observed 5:(4Δ+*T*) pentaborate(1-) anion, [B_5_O_6_(OH)_4_]^−^, but is additionally double deprotonated. The related compound Rb_2n_[Ga{B_5_O_10_}]_n_·4nH_2_O (**28**) has also been reported [25]. Here, the 5:(4Δ+*T*) building block is deprotonated 4 times to form the [B_5_O_10_]^5−^ anion with four of these anions coordinated to a tetrahedral 18-electron Ga^(III)^ center in chain-like 2-D structures.

Compound **27** is unique and features octahedral In^(III)^ centers coordinated by a tridentate dien ligand (*fac*-) and three monodentate {B_5_O_8_(OH)_2_}^3−^ ligands which bridge to other In^(III)^ centers in such a way as to produce a ‘dimeric’ 1-D chain with oxidoborate *O*-bridges between two In^(III)^ centers (Figure 7b). The H-bonding interactions between adjacent chains leads to a supramolecular H-bonded network.

### 2.5. Manganese(II) Borates

One 15-electron five-coordinate trigonal-bipyramidal Mn^(II)^ complex with a coordinated oxidoborate ligand has been reported, K_7_[(BO_3_)Mn{B_12_O_18_(OH)_6_)}]·H_2_O (**29**) [27]. The structure of this will be discussed in Section 2.7 since **29** is isostructural with an analogous 20-electron Zn^(II)^ complex and forms part of a family of structurally related of Zn^(II)^ compounds.

### 2.6. Nickel(II) Borates

The recently synthesized salt [Ni(H_2_O)_6_]_2_[NO_3_]_2_·[Ni(H_2_O)_4_{B_3_O_3_(OH)_4_}_2_]·2H_2_O (**30**) [14] is isostructural with **7**. The two [B_3_O_3_(OH)_4_]^−^ ligands in the neutral [Ni(H_2_O)_4_{B_3_O_3_(OH)_4_}_2_] complex are *trans* on octahedral 20-electron Ni^(II)^ centers and are coordinated by hydroxy *O*-donors bound to tetrahedral boron atoms of the triborate(1-) anion.

**Figure 8:**
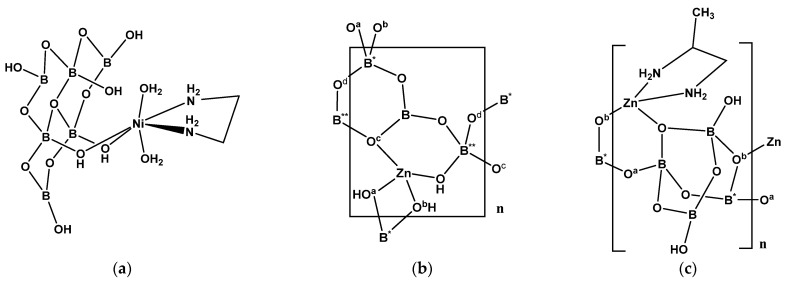
(**a**) The uncharged complex of [Ni(en)(H_2_O)_2_{B_6_O_7_(OH)_6_}]·H_2_O (**31**) showing the bidentate nature of the [B_6_O_7_(OH)_6_]^2−^ ligand, (**b**) the building unit of [Zn{B_3_O_4_(OH)_3_}]_n_ (**35**), and (**c**) the building unit of [Zn(pn){B_4_O_6_(OH)_2_}]_n_ (**36**).

Two Ni^(II)^ hexaborate(6-) complexes have recently been synthesized during the review period: [Ni(en)(H_2_O)_2_{B_6_O_7_(OH)_6_}]·H_2_O (**31**) [28] and [Ni(dmen)(H_2_O){B_6_O_7_(OH)_6_}]·5H_2_O (**32**) [28]. These compounds are neutral molecules and contain the [B_6_O_7_(OH)_6_]^2−^ ligand as described earlier for **4**, **10, 11** and **15–19** (Section 2.1, Section 2.2 and Section 2.3). These ligands are also found in Zn^(II)^ chemistry (Section 2.7). Both **31** and **32** are octahedral about the 20-electron Ni^(II)^ centers but differ in the denticity of the hexaborate(2-) ligands which are bidentate in **31** (Figure 8a) and tridentate in **32**. Both compounds demonstrate numerous intermolecular and intramolecular solid-state structure directing H-bond interactions.

The derivatized hexaborate(2-) ligand, with three 2-amino ethoxy groups, in place of the three -OH groups on the tetrahedral boron centers, functions as a hexadentate ligand in the 20-electron Ni^(II)^ complex, [Ni(NH_2_CH_2_CH_2_O)_3_{B_6_O_7_(OH)_3_}] (**33**) [18]. Compound **33** is isostructural with **12**.

### 2.7. Zinc(II) Borates

There have been more oxidoborate complexes reported for Zn^(II)^ than for any other metal. The coordinated oxidoborate ligands range in size from triborate(1-) to dodecaborate(6-).

The recently synthesized salt [Zn(H_2_O)_6_]_2_[NO_3_]_2_·[Zn(H_2_O)_4_{B_3_O_3_(OH)_4_}_2_]·2H_2_O (**34**) [14] is isostructural with **7** and **30**. The two [B_3_O_3_(OH)_4_]^−^ ligands in the neutral [Zn(H_2_O)_4_{B_3_O_3_(OH)_4_}_2_] complex are *trans* on octahedral 22-electron Zn^(II)^ centers and are coordinated by hydroxy *O*-donors bound to tetrahedral boron atoms.

Structural characterization of the commercially important Zn^(II)^ triborate, [Zn{B_3_O_4_(OH)_3_}]_n_ (**35**), revealed that it was a crosslinked 1-D coordination chain polymer of Zn^(II)^ [29]. The oxidoborate forms a 1-D polymeric chain (Figure 8b) and each 18-electron Zn^(II)^ center is coordinated by two oxygen atoms from two adjacent monomeric unit of the chain, and completes its tetrahedral arrangement by coordination from two hydroxide *O*-donors from a neighboring chain to crosslink the structure into a 2-D network. The network also has numerous interchain H-bond interactions. Each triborate(2-) FBB unit is a 6-membered boroxole ring with one {BO_3_} and two {BO_4_} units, i.e., 3:(Δ+2*T*).

Tetraborate ligands based on the 4(4-1) FBB are represented by the following compounds [Zn(pn){B_4_O_6_(OH)_2_}]_n_ (**36**) [30], [Zn(dap)_0.5_(dap’)_0.5_{B_4_O_6_(OH)_2_}]_n_·nH_2_O (**37**) [30], and [Zn(dab)_0.5_(dab’)_0.5_{B_4_O_6_(OH)_2_}]_n_·nH_2_O (**38**) [31]. Compounds **36-38** have identical Zn^(II^)/ligand/borate stoichiometries with similar, but non-identical, structures. All three contain tetrahedral 18-electron Zn^(II)^ centers with two *N*-donor amine and two *O*-donor tetraborate ligands, with the latter condensed into 1-D tetraborate chains. Compound **36** contains a chelating pn ligand (Figure 8c) whereas **37** (and **38)** has two dap (or dab) ligands on each Zn^(II)^ center both bridging other Zn^(II)^ centers and forming a 1-D coordination polymer chains in crosslinked inorganic–organic 2-D layered structures.

The FBB of 5:(4Δ+*T*) is present in [Zn(tren){B_5_O_7_(OH)_3_}] (**39**) [32] and this ligand is identical to that found in **9** (Section 2.2). [Zn(dab)_0.5_(dab’)_0.5_{B_5_O_7_(OH)_3_}]_n_ (**40**) [8] and [Zn(appip){B_4_O_6_(OH)(OB{OH}_2_})]_n_·3nH_2_O (**41**) (appip = *trans*-1,4-bis(3-aminopropyl)piperazine) [30] are based on a FBB of 4 with a pendant -OB(OH)_2_ replacing a hydroxyl group on a tetrahedral boron center of the tetraborate moiety with a 5:[4:(2Δ+2*T*)+Δ] framework and are isostructural with **1** (Section 2.1).

**Figure 9:**
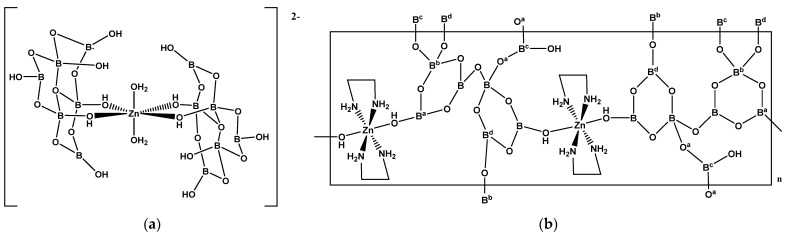
Drawings of **(a**) the insular anion in (NH_4_)_2_[Zn(H_2_O)_2_{B_6_O_7_(OH)_6_}_2_]·2H_2_O (**43**), and (**b**) the repeating unit of [{Zn(en)_2_{B_7_O_10_(OH)_3_}_2_]_n_ (**46**).

The 6:(3Δ+3*T*) FBB, described in Section 2.1, Section 2.2, Section 2.3 and Section 2.6, is also observed as a hexaborate(2-) ligand in Zn^(II)^ complexes. The following complexes have been prepared [Zn(dien){B_6_O_7_(OH)_6_}]·0.5H_2_O (**42**) [33], (NH_4_)_2_[Zn(H_2_O)_2_{B_6_O_7_(OH)_6_}_2_]·2H_2_O (**43**) [33], [Zn(en){B_6_O_7_(OH)_6_}]_n_·2nH_2_O (**44**) [34] and [Zn(pn){B_6_O_7_(OH)_6_}]_n_·1.5nH_2_O (**45**) [34]. The 22-electron Zn^(II)^ centers in **42**-**45** are all octahedral with *N*_3_*O*_3_, *O*_6_, *N*_2_*O*_4_ and *N*_2_*O*_4_ donor sets, respectively. The hexaborate(2-) ligands in **42**, **44** and **45** are tridendate but are bis(bidentate) and *trans* in the centrosymmetric anion in **43** (Figure 9a). Compounds **44** and **45** are 1-D coordination polymers with a -OH group of a trigonal boron of the coordinated hexaborate(2-) anion bridging onto another Zn^(II)^ center. This configuration has also been observed in Cd^(II)^ (**4**, Section 2.1) and Cu^(II)^ (**15** and **16**, Section 2.3) chemistry.

{{Zn(en)_2_B_7_O_10_(OH)_3_}_2_]_n_ (**46**), also formulated as [Zn(en)_2_{B_7_O_12_(OH)}] in Ref. [9], is a 3-D coordination polymer comprised of an octahedrally coordinated 22-electron Zn^(II)^ center based on square planar [Zn(en)_2_] units axially coordinated by a *O*-donor condensed oxidoheptaborate framework. The oxidoborate FBB in **46**, [B_7_O_10_(OH)_3_}]_n_^2n−^, can be described by a compounded descriptor 7:[(3:2Δ+*T*)+(3:2Δ+*T*)+Δ] with triangular BO_2_(OH) cross-linking units (Figure 9b).

**Figure 10:**
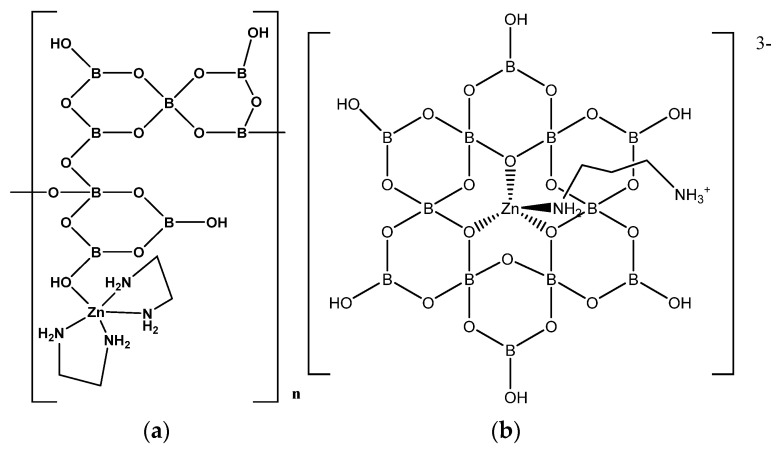
(**a**) The repeating unit of [Zn(en)_2_{B_8_O_11_(OH)_4_}]_n_ (**47**), and (**b**) a drawing of the [(Hdap)Zn{B_12_O_18_(OH)_6_}]^3−^ anion in (H_2_dap)_3_[(Hdap)Zn{B_12_O_18_(OH)_6_}]_2_· 14H_2_O (**51**).

[Zn(en)_2_{B_8_O_11_(OH)_4_}]_n_ (**47**) [10] was reported in 2017 and has a similar stoichiometry to **3** but is isomeric, with a different oxidoborate condensation mode and a 1-D polymer chain. (Figure 10a). Nevertheless, the octaborate(2-) anion is best designated by the compounded descriptor 8:[5:(4Δ+*T*)+3:(2Δ+*T*)].

[Zn_2_(dap)(dap’){B_8_O_13_(OH)_2_}]_n_ (**48**) [30] has a similar structure to **37** and **38** with the 18-electron Zn^(II)^ centers tetrahedral with 2 monodentate bridging dap *N*-donor ligands. The octaborate(4-) *O*-donor ligands are based on two condensed tetraborate(2-) building blocks via an O bridge, 8:{4(2Δ+2*T*)}_2_. However, this now results in 2-D layers rather than 1-D chains and the 2-D layers are further crosslinked into 3-D networks, by the amines.

The large insular anion [B_12_O_18_(OH)_6_]^6−^ has been reported in K_7_[(BO_3_)Zn{B_12_O_18_(OH)_6_)}]·H_2_O (**49**) [35], [(Hen)Zn{B_12_O_18_(OH)_6_}Zn(en)(Hen)]·8H_2_O (**50**) [36] and (H_2_dap)_3_[(Hdap)Zn{B_12_O_18_(OH)_6_}]_2_·14H_2_O (**51**) [33]. The dodecaborate(6-) anion is based on a hexameric FBB of 3 i.e., 12:{3:(Δ+2*T*)}_6_, with all tetrahedral boron centers linking boroxole {B_3_O_3_} rings, generating a larger inner 12-membered B/O alternating B_6_O_6_ ring. Stereochemically, three of these potential *O*-donor atoms point to one side of the large ring and three face towards the other side. The anion in **51**, [(Hdap)Zn{B_12_O_18_(OH)_6_}]^3−^, which is typical of the oxidoborate building blocks contained within these structures, has a tetrahedral 18-electron Zn^(II)^ center coordinated by *fac O*-donors from the dodecaborate(6-) ligand and a monodentate *N*-donor from a protonated 1,3-diamminopropane ligand (Figure 10b). There is extensive H-bonding between anions forming a supramolecular 3-D H-bonded lattice.

## 3. Synthetic Methods

### 3.1. Slow Crystallization by Solvent Evaporation

This method involves slow crystallization from aqueous or a miscible aqueous/organic solution which originally contained B(OH)_3_ and metal precursor complexes. The oxidoborate compounds are templated by the transition-metal/p-block metal complexes present in the dynamic combinatorial library (DCL) of oxidoborate anions that are present in solution in equilibrium concentrations [37]. Products are generally under thermodynamic control especially when metal-ligand exchange equilibria (and hydroxyoxidoborate-H_2_O equilibria) are fast. The solvents used for evaporation in the boric acid solution are often H_2_O or H_2_O/EtOH or H_2_O/MeOH. Crystallization of the product may take a few hours to several weeks. This is illustrated for the preparation of [Cu(en){B_6_O_7_(OH)_6_}]_n_·3nH_2_O (**16**) from [Cu(en)_2_]SO_4_ and B(OH)_3_ [20]. [Cu(en)_2_]SO_4_ (3.6 mmol) and BaSO_4_·8H_2_O (3.6 mmol) were dissolved in H_2_O (20 mL). The solution was stirred for 20 min at room temperature and the precipitate that formed (BaSO_4_) was removed by filtration. B(OH)_3_ (25 mmol) was added to the filtrate which was then stirred for 40 min. The resulting solution was left in several small vials to crystallize. After standing for 35 days the product **16** was collected by filtration as blue crystals in 31% yield. Compounds **11**, **13**–**19**, **31**, **32**, **35, 42**–**45**, **50** and **51** were prepared by a method similar to this. Oxidopolyborates prepared by this method are often insular salts and are often less condensed than those prepared by the methods described below, which generally use more forcing conditions.

### 3.2. Solvothermal/Hydrothermal Methods

Many organic-inorganic oxidoborates described in this article are synthesized by solvothermal (or hydrothermal) methods. In this method the non-aqueous solvent (or H_2_O) is placed in a sealed reaction vessel together with boron containing materials, transition-metal/p-block metal salts and organic ligands and heated to a specific temperature (usually 100–250 °C) for a set time period. Solvothermal reactions can lead to metastable, kinetically controlled, products. This method is illustrated by the preparation of [Cd(dap)_0.5_(dap’)_0.5_{B_5_O_8_(OH)}]_n_·nH_2_O (**2**) [9]. H_3_BO_3_ (5 mmol) and Cd(NO_3_)_2_·4H_2_O (1 mmol) were slowly added to a *N*,*N*-dimethylformamide (2 mL) and 1,3-dap (1 mL) solution with stirring. The white emulsion was then sealed in a Teflon-lined 25 mL autoclave reactor and heated at 180 °C for 7 days. After cooling to room temperature, crystals of **2** (36% yield based on H_3_BO_3_) were collected by filtration, washed with H_2_O, and dried in air. Compounds **1–6, 8, 9, 12, 20–29, 33, 36–41** and **46–49** were prepared by this general method. This relatively low temperature hydrothermal method is popular among many researchers since the necessary autoclave reactors are widely available in both academic and industrial laboratories.

### 3.3. Molten Salt Methods

In this method a molten salt (120–250 °C) is used as both solvent and reactant and the reaction vessel is charged with B(OH)_3_ and any other necessary reaction materials. This is illustrated by the preparation of [Co(H_2_O)_6_]_2_[NO_3_]_2_·[Co(H_2_O)_4_{B_3_O_3_(OH)_4_}_2_]·2H_2_O (**7**) [14]. Co(NO_3_)_2_·6H_2_O (1 mmol) was placed in a 25 mL beaker and heated in an oil bath at 120 °C until fully molten (*ca.* 1 min). H_3_BO_3_ (0.8 mmol) was then added, and the mixture was stirred until the H_3_BO_3_ was completely dissolved (*ca*. 30 min). The solution was then allowed to cool to room temperature and left to crystallize. After 14 days, transparent colorless crystals of **7** (30% based on H_3_BO_3_) were deposited on the bottom of the beaker. Compound **7** was isolated by filtration. Compounds **30** and **34** were also prepared by this method.

## 4. Physical, Spectroscopic Properties and Potential Applications

The oxidoborate complexes described in this manuscript are all crystalline solids with high melting (or decomposition) points. Techniques used to characterize the oxidoborates and to study their possible applications include structural studies (single-crystal XRD, powder-XRD, TEM), optical properties (diffuse reflectance spectroscopy, nonlinear optical studies (NLO), photoluminescence), magnetic properties, vibrational spectroscopy, thermal studies and catalytic investigations.

Oxidoborate complexes derived from transition-metals are generally colored but the *d*^10^ ions (Cd^(II)^, Zn^(II)^, Ga^(III)^ and In^(III)^) are colorless. UV/Vis absorption properties of several oxidopolyborate complexes (**1**, **4**, **5**–**8**, **22**–**24**, **28**, **30**, **34**, **36**, **37**, **40**, **41**) have been studied by diffuse reflectance spectroscopy and band-gaps with energies of ranging from 3.1 eV for [(Cd_3_){B_6_O_9_(OH)_2_}_2_(NO_3_)_2_(H_2_O)_4_]_n_ (**5**) to 5.9 eV for [Cd(dab)_0.5_(dab’)_0.5_{B_5_O_7_(OH)_3_}]_n_ (**1**) have been noted.

The band-gaps of [Cd(pn){B_6_O_7_(OH)_6_}]_n_·nH_2_O (**4**), [Ga(teta){B_5_O_8_(OH)_2_}]_n_·nH_2_O (**23)** and [In(teta){B_5_O_8_(OH)_2_}]n·1.5nH_2_O (**24**) show interesting but different temperature effects. Compounds **23** and **24** exhibited blue maximum luminescence at 446 and 472 nm, respectively, when excited at 352 and 342 nm, respectively. The luminescence intensity of these two compounds increased with a decreasing temperature and was at its most intense at 80 K. Compound **4** exhibited the maximum luminescence at 444 nm with a 360 nm excitation light source, and the luminescence intensity increased with the decrease of temperature to 230 K where it was at its most intense. The luminescence intensity of **4** decreased when the temperature was further lowered to 80 K [11]. Compounds [M(dab)_0.5_(dab’)_0.5_{B_5_O_7_(OH)_3_}]_n_ (M = Cd, **(1**); M = Zn (**40**)) have been shown to display blue luminescence with maximum fluorescent emission at 423 and 412 nm, when excited at and 356 or 384 nm, with lifetimes of 4.67 and 4.09 ns, for **1** and **40**, respectively [8]. It is reported that the blue luminescence of two compounds originates from their inorganic oxidoborate frameworks.

Nonlinear optical (NLO) effects are also displayed by the following hybrid metal oxidoborates Rb_2n_[Ga{B_5_O_10_}]_n_·4nH_2_O (**28**) [25], K_7_[(BO_3_)Mn{B_12_O_18_(OH)_6_)}]·H_2_O (**29**) [27], [Zn(pn){B_4_O_6_(OH)_2_}]_n_ (**36**) [30] and [Zn(appip){B_4_O_6_(OH)(OB{OH}_2_})]_n_·3nH_2_O (**41**) [30]. For example, compound **41** belongs to the space group *P2_1_*, and since this point group is non-centrosymmetric **41** might be expected to exhibit second order nonlinear optical effects; indeed, powdered **41** does exhibit SHG behavior with a response of 29 mV using a laser of beam energy with 2.83 mJ/pulse [30]. Compound **36** had a response of 24 mV while the response of the SHG standard, KH_2_PO_4_ (KDP), was 175 mV under similar conditions [30]. Compound **28** crystallizes in chiral space group *C222_1_*. SHG measurements on a Q-switched Nd:YAG laser with sieved powdered samples (70−100 mesh) revealed that compound **28** displays a moderately strong SHG response approximately equal to that KDP [25]. The SHG response of **29** was again moderate and equal to that of KDP [27].

Many of the compounds described within this review are diamagnetic but those containing Cu^(II)^ (**13**–**21**) and Ni^(II)^ (**31**–**32**) centers are paramagnetic. The magnetic properties of the Ni^(II)^ complex **30** have not been reported. Magnetic properties of the Co^(II)^ (**7**–**12**) and the Mn^(II)^ (**29**) oxidoborate complexes were also not reported, although these would be expected to be paramagnetic. The μ_eff_ values (per Cu^(II)^ atom) of the multi-metallic complexes [H_3_O]_4_[Cu_7_(NH_3_)_2_(H_2_O)_4_{B_24_O_39_(OH)_12_}]·13H_2_O (**13**), H_6_[Cu_4_O{B_20_O_32_(OH)_8_}]·25H_2_O (**20**) and H_6_[Cu_4_O{B_20_O_32_(OH)_8_}]·34H_2_O·8B(OH)_3_ (**21**) are much lower than expected by the spin-only formula and **20** and **21** are temperature dependent and display anti-ferromagnetic behavior [24].

A complex borate, [Ni(en)_3_]_n_[Hen]_n_[B_9_O_13_(OH)_4_]_n_·nH_2_O, containing isolated cations and a partially condensed anionic a one-dimensional oxidoborate chain has ferroelectric properties at room temperature [38]. The electrical hysteresis loop was observed when an electric field between −28 and +28 kV was applied to the sample. Spontaneous polarization (*P_s_* of about 52 nCcm^−2^) also occurred during the measurement with remnant polarization (*P_r_* of about 30 nCcm^−2^) and a coercive field (*E_c_*) of about 28 kVcm^−1^. The *P_s_* of this compound is very close to that of KH_2_PO_4_^−^ type ferroelectrics. The related compound, [Ni(en)_2_(pip)][B_5_O_6_(OH)_4_]_2_, which contains insular oxidoborate anions rather than coordinated oxidoborate ligands, was reported as the first templated oxidoborate with ferroelectric properties [39]. The *P_s_* value for [Ni(en)_2_(pip)][B_5_O_6_(OH)_4_]_2_ of 20 nCcm^−2^ for is significantly higher than that of typical organic ferroelectric compounds, e.g., β-quinol-methanol (*P_s_* = 6 nCcm^−2^). A related pentaborate derivative, [Zn(dab)_0.5_(dab’)_0.5_{B_5_O_7_(OH)_3_}]_n_ (**40**), has been prepared more recently, and exhibits a wide *E_c_* value of ca. 6 kV cm^−1^ to 11 kV cm^−1^ [8].

Vibrational (IR) data are commonly reported for oxidoborate complexes. In addition to diagnostic bands associated with organic ligands (O-H, N-H, C-C, C-N, C-C etc. stretches and bends) strong absorptions associated with ligand-M and B-O stretches (1400–1000 cm^−1^) are often observed but usually are not diagnostic since they are in the IR fingerprint region. In general, B-O stretching modes can be subdivided into four specific regions (asymmetric stretches: B_trig_-O, 1450–1300 cm^−1^; B_tet_-O, 1150–1000 cm^−1^; symmetric stretches: B_trig_-O, 960–890 cm^−1^, B_tet_-O, 890–740 cm^−1^) with bending and deformation modes at lower energy [40]. Nevertheless, Li and co-workers have tabulated diagnostic wavenumbers for specific smaller oxidoborate anions [40]. Hexaborate(2-) derivatives, with a FBB of 6:(3*D*+3*T*), are fairly common as oxidoborate ligands (e.g., compounds **4**, **5**, **10**–**12**, **15**–**20, 42**–**45**) and diagnostic bands at 955 and 808 cm^−1^ have been proposed for this anion [20,40]. Tentative diagnostic bands (1047, 952, 902 and 857 cm^−1^) have also been reported for icosaborate(6-) derivatives (**49**–**51**) [33].

The most studied property of inorganic–organic hybrid borates is their thermal behavior. Indeed, all compounds described within this review, except **10, 12, 33** (which are reported in crystallographic journals [16,18]), have their thermal properties documented. Many of the thermal studies have been undertaken in air from room temperature up to 800 °C, although a few (**4, 23, 24**) report data obtained through heating under N_2_ to a similar temperature. Generally, thermal decomposition is a multistage process which includes a low temperature loss of interstitial molecules (where present), a moderate temperature dehydration and cross-linking of oxidoborate hydroxyl groups, and a high temperature oxidation process (or removal of volatile organics) to generate a glassy borate residual solid. This solid can be formulated as an anhydrous metal borate (i.e., a mixed metal/boron oxide) with a M:B ratio that maintains the M:B ratio of the original oxidoborate complex. These principles are illustrated for compounds [Cd(pn){B_6_O_7_(OH)_6_}]_n_·nH_2_O (**4**)**,** [H_3_O]_4_[Cu_7_(NH_3_)_2_(H_2_O)_4_{B_24_O_39_(OH)_12_}]·13H_2_O (**13**) and [Zn(en){B_6_O_7_(OH)_6_}]_n_·2nH_2_O (**44**). Compound **44** decomposes thermally (in air) by the loss of 2 interstitial H_2_O (100–180 °C), condensation of hydroxyoxidoborate with loss of 3H_2_O (180–320 °C) and oxidation of the en ligand (320–470 °C) to leave as a residue ZnB_6_O_10_ (= ZnO·3B_2_O_3_) [34]. Compound **13** thermally decomposes in air by the loss of 21 (interstitial and coordinated) H_2_O and 4 (coordinated) NH_3_ molecules (at 70–160 °C) and condensation of hydroxyoxidoborate groups with loss of 8H_2_O (at 160–300 °C) to afford Cu_7_B_24_O_43_ (= 7CuO·12B_2_O_3_) [19]. Occasionally, the individual processes overlap but the endpoint is the same. Thus, **4** losses weight continuously in one step upon heating, under N_2_, from room temperature to 800 °C [11]. In this process the guest water molecules are removed together with pn ligands and the oxidopolyborate fully condenses to afford CdB_6_O_10_ (= CdO·3B_2_O_3_) [11]. Non-metal cation polyborates have been considered to be thermal precursors to porous materials [5]. To date this has not been successful [41,42,43] but recent studies on H_6_[Cu_4_O{B_20_O_32_(OH)_8_}]·25H_2_O (**20**) and H_6_[Cu_4_O{B_20_O_32_(OH)_8_}]·34H_2_O·8B(OH)_3_ (**21**), which contain large 3-D intersecting channel systems with PLATON calculated solvent accessible voids of ca. 60%, suggest that mesoporous materials may be available from hybrid organic oxidoborates [24].

Fire retardancy is often linked to a compound’s thermal properties and [Zn{B_3_O_4_(OH)_3_}]_n_ (**35**) [31] is well known for this property and has been commercially exploited. Many zinc borates also possess such properties [44] and the recently prepared hybrid oxidoborate complex [Zn(H_2_O)_6_]_2_[NO_3_]_2_·[Zn(H_2_O)_4_{B_3_O_3_(OH)_4_}_2_]·2H_2_O (**34**) [14] has also been reported as a promising flame retardant, at 15 wt.% loading, for ABS (acrylonitrile butadiene styrene), an important engineering thermoplastic material.

Hybrid metal-organic oxidoborates have been used as precursors to thermally prepared catalysts [9] e.g., carbon material catalysts derived from thermal treatment of [Cd(dap)_0.5_(dap’)_0.5_{B_5_O_8_(OH)}]_n_·nH_2_O (**2**) and [{Zn(en)_2_{B_7_O_10_(OH)_3_}_2_]_n_ (**46**) have been used to electrochemically reduce CO_2_ to CO. Initial results indicate that the oxidoborate is a useful catalyst precursor since at 1.4V CO formation is 51% higher than that of RHE. This may be a promising development as a low-cost alternative to traditional noble metal catalysts.

## 5. Conclusions

Since metals generally have multiple coordination sites, they often can form coordination linkages with organic (*N*-donor) and oxidoborate (*O*-donor) ligands. These organic/inorganic oxidopolyborate hybrid complexes are structurally diverse and often have polymeric structures based on unique frameworks. The hybrid organic oxidoborate materials in such integrated structures can afford materials with unique properties and which may overcome possible deficiencies inherently present in related single component systems. Hybrid inorganic−organic oxidoborates have attracted considerable recent research attention, and interesting properties such as photoluminescence, ferroelectric and NLO and catalytic properties have been discovered and these are summarized within the review. Initial results indicate that these properties can be tailored and manipulated by adjusting the organic ligands, the oxidoborate motif and/or the central metallic ion, and that such compounds are a promising area for future research.

## Data Availability

Not applicable.

## Abbreviations

appip	*trans*-1,4-bis(3-aminopropyl)piperazine
dab	1,4-diaminobutane
dap	1,3-diaminopropane
deen	*N*,*N*-diethyl-1,2,-diaminoethane
dmen	*N*,*N*-dimethyl-1,2-diaminoethane
en	1,2-diaminoethane
pip	piperidine
pn	1,2-diaminopropane
py	pyridine
teta	tetraethylenetriamine
tren	tris(2-aminoethyl)amine
tmeda	*N*,*N*,*N’*,*N’*-tetramethyl-1,2-diaminoethane
